# Sociodemographic inequities in cervical cancer screening, treatment and care amongst women aged at least 25 years: evidence from surveys in Harare, Zimbabwe

**DOI:** 10.1186/s12889-019-6749-6

**Published:** 2019-04-24

**Authors:** O. Tapera, W. Kadzatsa, A. M. Nyakabau, W. Mavhu, G. Dreyer, B. Stray-Pedersen, Hendricks SJH

**Affiliations:** 1 University of Pretoria, School of Health Systems and Public Health, Pretoria, South Africa; 2 Parirenyatwa Group of Hospitals, Radiotherapy Centre, Harare, Zimbabwe; 3 Centre for Sexual Health & HIV/AIDS Research (CeSHHAR), Harare, Zimbabwe; 4 Department of Obstetrics and Gynaecology, University of Pretoria, Gynaecologic Oncology, Pretoria, South Africa; 5 Institute of Clinical Medicine, University in Oslo and Womens’ Clinic, Oslo University Hospital, Oslo, Norway; 6 Sefako Makgatho Health Sciences University, Pretoria, South Africa; 7 University of Fort Hare, East London, South Africa

**Keywords:** Cervical cancer, Inequity, Socio-demographic, Screening, Access, Treatment, Stratified random sampling, Harare

## Abstract

**Background:**

Cervical cancer is the most commonly diagnosed cancer among women in Zimbabwe; however; access to screening and treatment services remain challenged. The objective of this study was to investigate socio-demographic inequities in cervical cancer screening and utilization of treatment among women in Harare, Zimbabwe.

**Methods:**

Two cross sectional surveys were conducted in Harare with a total sample of 277 women aged at least 25 years. In the community survey, stratified random sampling was conducted to select 143 healthy women in Glen View, Cranborne, Highlands and Hopely communities of Harare to present high, medium, low density suburbs and rural areas respectively. In the patient survey, 134 histologically confirmed cervical cancer patients were also randomly selected at Harare hospital, Parirenyatwa Hospital and Island Hospice during their routine visits or while in hospital admission. All consenting participants were interviewed using a validated structured questionnaire programmed in *Surveytogo* software in an android tablet. Data was analyzed using *STATA* version 14 to yield descriptive statistics, bivariate and multivariate logistic regression outcomes for the study.

**Results:**

Women who reported ever screening for cervical cancer were only 29%. Cervical cancer screening was less likely in women affiliated to major religions (*p* < 0.05) and those who never visited health facilities or doctors or visited once in previous 6 months (p < 0.05). Ninety-two (69%) of selected patients were on treatment. Women with cervical cancer affiliated to protestant churches were 68 times [95% CI: 1.22 to 381] more likely to utilize treatment and care services compared to those in other religions (*p* = 0.040). Province of residence, education, occupation, marital status, income (personal and household), wealth, medical aid status, having a regular doctor, frequency of visiting health facilities, sources of cervical cancer information and knowledge of treatability of cervical cancer were not associated with cervical cancer screening and treatment respectively.

**Conclusion:**

This study revealed few variations in the participation of women in cervical cancer screening and treatment explained only by religious affiliations and usage of health facilities. Strengthening of health education in communities including churches and universal healthcare coverage are recommended strategies to improve uptake of screening and treatment of cervical cancer.

## Background

Cervical cancer is the most commonly diagnosed cancer amongst women and accounts for 32% of all cancers in Zimbabwe yet it is preventable [1]. At least 4.5 million women aged at least 15 years risk contracting cervical cancer in their life time in the country [2]. Recent data has shown that a total of about 7000 cases of cervical pre-cancers and cervical cancer (2270 cases) are diagnosed and about 1451 deaths are recorded annually [2, 3]. Cervical cancer burden is increasing in Zimbabwe, predominantly due to high prevalence of HIV/AIDS and limited screening and treatment services [4]. The past few years have seen increases in the number of cervical cancer screening facilities offering free services across the country especially in urban areas; however these services remain limited in rural areas where 66% of the women reside [4, 5]. Zimbabwe conducted an HPV vaccination pilot intervention which started in 2014 and was targeted at girls 10–14 years in schools to reduce the risk of cervical cancer [4]. However, given the impracticalities of covering the entire female population with vaccination the nation will continue relying on cervical cancer screening and treatment of pre-cancerous lesions to prevent the disease. In addition, cervical cancer diagnosis, staging and treatment remain centralized and the associated direct and indirect costs remain unaffordable to the majority of patients [4, 6, 7]. Inequities in cervical cancer screening and treatment in Zimbabwe have been reported anecdotally but not much is known on their magnitude and influencing socioeconomic factors.

The United Nations Declaration of Human rights Article 25 states that: “Everyone has the right to a standard of living adequate for the *health* and well-being of himself and of his family, including food, clothing, housing and *medical care* and necessary social services” [8]. In pursuance of this international declaration Zimbabwe’s health policies have been developed to ensure equity over the years. The National Cancer Strategy in Zimbabwe (2013–2017) outlined equity as its number one guiding principle yet the policy did not indicate measures meant to ensure equity in access of cancer preventive and control measures [9]. Another recent policy, the Zimbabwe Cervical Cancer Prevention and Control Strategy (2016–2020) outlined equity as a key component to ensure equitable distribution of cervical cancer prevention and treatment services [10]. Disparities in healthcare access and utilization entrenched by age, social class, income, occupation, ethnicity, gender and place of residence have been documented [11].

Several studies have reported factors associated with uptake of cervical cancer screening in different contexts. The main factors reported were: education, income levels, marital status, age, employment and area of residence [12–24]. However in Zimbabwe, some researchers found that financial independence among women and living in mining and resettlement areas were associated with uptake of cervical cancer screening [24]. Studies from across the world have also reported different determinants of cancer treatment uptake and these included: age, ethnicity, socioeconomic class, income, health insurance status, marital status, area of residence [25–29]. One study in USA contrary to the researchers’ hypothesis, reported that socio-demographic factors such as race/ethnicity, insurance status, and socioeconomic status, did not influence the receipt of radiation therapy for rectal cancer [30]. There is a knowledge gap regarding inequities in the uptake of cervical cancer screening and treatment in Zimbabwe. Our present work was an imperative starting point to building evidence in the developing context for use in policy and design of interventions.

## Methods

### Data sources

Two cross sectional surveys were conducted in Harare between January and April 2018 to gather data for the study. The first survey was community based, where 143 women aged at least 25 years were randomly selected from stratified communities. Based on the Dopson sample size calculator [3, 31] the minimum sample required for the survey was 140 participants. Harare communities were stratified into low, medium and high density and rural areas and probability proportional to size sampling was conducted to determine the subsample for each stratum using census data [5]. Using the ballot approach one community was randomly selected from the list of communities or suburbs that had been grouped into their respective strata based on City of Harare data. The selected suburbs were Glen View (high density), Cranborne (medium density), Highlands (low density) and Hopely (rural area). The subsamples achieved were 31 for each suburb and 50 for the rural area which exceeded the required minimum sample size of 140, thereby improving on the precision of estimates. In communities, households were selected randomly by selecting a reference household and going around in an anti-clockwise direction maintaining a sampling interval of 7–10 households in high density and rural area and 3–5 households in medium and low density suburbs. At household level, when more than one woman was eligible for participation, the Kish grid approach [32] was used to select a respondent in that household. Interviews were conducted after obtaining written consent from the potential participant.

For patient survey, histologically confirmed cervical cancer patients or survivors at least 25 years old were selected using health facility records. All confirmed cervical cancer patients or survivors who visited participating health facilities between February and April 2018 were considered for participation in the study after obtaining written consent. The subsamples for Harare Hospital, Parirenyatwa Hospital and Island Hospice were 36 (27%), 86 (64%) and 12 (9%) respectively and these were proportional to the flow of cervical cancer patients based on 2017 data from the facilities. The minimum sample size required for the patient survey was 80, and this was based on the flow of patients in 2017. However, a total of 134 participants were enrolled in the study to increase the precision of estimates from the study given the different disease stages, treatments and backgrounds of the patients. Out and in-patients from Harare Central Hospital, Parirenyatwa Hospital and Island Hospice in Harare were eligible to participate in the study after consenting in writing. The researchers sought the assistance of health workers in the participating health facilities to identify histologically confirmed cervical cancer patients and to confirm treatment. During the data collection period, health workers were sensitized about the study. Patients were sensitized by their health workers and asked if they would want to participate in the study. Patients who agreed verbally to participate were then referred to the researchers for informed consent and interviews. This process was done for outpatients as well as in-patients. The researchers also verified the diagnoses of the patients during the interviews to ensure that only eligible participants were enrolled for the study. Patients were selected regardless of their place of residence. Patients who were terminally ill, mentally challenged or confused and refused to consent were not eligible to participate in the study.

### Data collection methods

Both the community and patient surveys used a similar validated structured questionnaire [33], administered by the researchers in the most appropriate language to the participant, which was either English or Shona. This approach allowed for probing and verification of issues during the interview process. Survey data were collected electronically using android tablet programmed with *SurveyToGo* software. This software saved the collected data automatically in a cloud server and the researchers downloaded the data files in *csv format* from the server using a laptop. The *CSV* data file was imported into *STATA* software which was used for data cleaning and analyses. The use of electronic data collection methods allowed the researchers to review data in real-time and collection of geo-coordinates for verification and monitoring sampling processes in the field.

### Variables

The socioeconomic equity variables used in the models were province of residence categorized as Manicaland, Masvingo, Midlands, Mashonaland Central, Mashonaland East, Mashonaland West and Harare, education (primary, secondary, higher and none), religion (Roman Catholic, Protestant, Pentecostal, Apostolic sect and Other), occupation (unemployed, professional, self-employed and other), marital status (married/co-habiting, never married, widowed and divorced or separated), personal income (no income, <US$200, 200–400 and ≥ 430), household income (no income, <US$600, 600–1000 and ≥ 1200) medical aid status (yes and no) and wealth (poor, middle and rich). The categories for the variables used in the models were based on the number of observations obtained and meaningfulness based on literature [12–30]. The selection of the variables in the model was also based on literature and validated tool used in the study [12–30, 33]. Confounding variables adjusted for in the logistic regression model were head of households’ occupation and education and participants’ area of residence (categorized as urban and rural areas) and these were based on literature [12–30]. The outcome variable for cervical cancer screening utilization was “*ever screened for cervical cancer*” and this referred to at least one screening session regardless of the method used. This variable was self-reported, though they were follow-up questions about name of facility and the date of the last screening to validate uptake of screening services. The outcome variable for cervical cancer treatment utilization was defined as any administered treatment modality: surgery, chemotherapy and radiotherapy used singly or in combination and these were established from medical records and health professionals.

### Data analysis

Univariate and bivariate analyses were conducted to yield descriptive statistics. Chi-squared test was used to determine significance of differences in proportions among different groups of participants. The groups were based on history of cervical cancer screening and treatment utilization at the time of the survey. Multiple logistic regression models were used to determine the socio-demographic factors associated with screening and utilization of treatment of cervical cancer using binary outcome variables. The logistic regression models were used to estimate the odds of uptake of screening and treatment services for cervical cancer by healthy women and women with cervical cancer respectively in each socioeconomic group. The models allowed us to identify the disparities in the uptake of screening and treatment services by healthy women and patients compared to reference groups respectively. A *p*-value of < 0.05 at 95% CI was considered statistically significant. Data analyses were conducted using *STATA* version 14 software (StataCorp LLC, Texas).

## Results

The mean age of participants in both the community and patient surveys was 43 years (SD = 13.4). The mean age of women who participated in the community survey was 35 years (SD = 8.6) while those who reported ever screening had a mean age for 37 years (SD = 9.1). The proportion of women in the community survey who reported ever being screened for cervical cancer was only 29%.The majority (79%) of the participants who reported ever screening for cervical cancer were less than 45 years of age. Eight-6 % of the community survey participants who reported ever screening for cervical cancer were from urban areas while 14% were from the rural area. By further disaggregating the urban areas, 48% of women were from low density, 29% from medium density and only 9% from the high density area. With regards to marital status, the majority (74%) of the women reporting ever screening for cervical cancer were married. Seventy-one percent of the women had secondary education and 93% of the heads of households with women who reported ever screening for cervical cancer had at least higher education. More than half (52%) of the participants who reported ever screening for cervical cancer were affiliated to Protestant and Pentecostal churches. Thirty-three percent of the women who reported ever screening for cervical cancer screening were unemployed. The proportions of women who ever screened for cervical cancer who had no personal and household income were 48 and 50% respectively. Eight 1 % of these women were poor while 60% reported being on medical aid (health insurance).

Table 1 shows that the mean age of women who received cervical cancer treatment was 53 years (SD = 12.7) and 45% of the patients were Harare residents. Fifty-one percent of the patients who were treated lived in urban areas compared to 49% who resided in rural areas. Close to half of the patients who received cervical cancer treatment (48%) were widows and 33% were married or had partners. More than half (54%) of the patient who had received treatment had secondary education while 46% of their household heads had at least higher education. Forty percent of the patients who were treated were household heads. The majority (65%) of the patients who were treated for cervical cancer were unemployed while 16% of their household heads were also unemployed. Fifty-seven percent and 55% of the treated patients had no personal and household income respectively. Only 24% of the patients who received treatment were on medical aid while 76% were not. Thirty-two percent of the treated patients were poor while more than half (52%) were rich.

**Table 1:** Distribution of healthy women and cervical cancer patient participants by socio-demographic characteristics

Participant type	Healthy women, *N* = 143			Cervical cancer patients *N* = 134		
Socio-demographic variables	[*N* = 143] (%)	Ever screened [*n* = 42] (%)	*p*-value	[*N* = 134] (%)	Treated [*n* = 92] (%)	*p*-value
Province of residence						
Manicaland	–	–	–	17 (12)	14 (15)	0.193
Masvingo	–	–	–	9 (7)	8 (9)	0.176
Midlands	–	–	–	7 (5)	5 (6)	0.871
Matebeleland North	–	–	–	1 (1)	1 (1)	0.498
Mashonaland Central	–	–	–	5 (4)	4 (4)	0.671
Mashonaland East	–	–	–	24 (18)	16 (17)	0.871
Mashonaland West	–	–	–	5 (4)	3 (3)	0.671
Harare	143 (100)	42 (100)	0.120	66 (49)	41 (45)	0.217
Residence						
Urban	93 (65)	36 (86)	**0.001**	74 (55)	47 (51)	0.154
Urban_Low density	31 (21.7)	20 (48)	**< 0.001**	3 (2)	3 (3)	0.237
Urban_High density	31 (21.7)	4 (9)	**0.023**	67 (50)	40 (44)	**0.025**
Urban_Medium density	31 (21.7)	12 (29)	0.197	4 (3)	4 (4)	0.170
Rural	50 (35)	6 (14)	**0.001**	60 (45)	45 (49)	0.154
Age (years)	Mean (35)	Mean (37)		Mean (52)	Mean (53)	
25–34	78 (55)	21 (50)	0.481	6 (4)	4 (4)	0.914
35–44	40 (28)	8 (19)	0.981	31 (23)	19 (21)	0.313
45–54	22 (15)	12 (29)	0.434	41 (31)	26 (28)	0.642
55 or more	3 (2)	1 (2)	0.879	56 (42)	43 (47)	0.180
Ethnicity						
Shona	133 (93)	39 (92.8)	0.964	130 (97)	88 (96)	0.170
Ndebele	6 (4)	1 (2.4)	0.485	2 (1)	2 (2)	0.336
Other	4 (3)	2 (4.8)	0.358	2 (2)	2 (2)	–
Marital status						
Married/co-habiting	98 (69)	31 (74)	0.381	52 (39)	30 (33)	0.029
Never married	17 (12)	3 (7)	0.258	1 (1)	1 (1)	0.498
Widowed	13 (9)	2 (5)	0.246	59 (44)	44 (48)	0.190
Divorced or separated	15 (10)	6 (14)	0.339	22 (16)	17 (18)	0.341
Religion						
Roman Catholic	24 (17)	7 (17)	0.981	34 (25)	24 (26)	0.779
Protestant	22 (16)	11 (26)	**0.021**	24 (18)	21 (23)	**0.028**
Pentecostal	56 (39)	11 (26)	**0.040**	34 (25)	21 (23)	0.316
Apostolic sect	27 (19)	5 (12)	0.169	34 (25)	24 (26)	0.779
Other	14 (9)	8 (19)	**0.016**	8 (7)	2 (2)	**0.006**
Education						
Primary	19 (13)	2 (5)	0.053	43 (32)	30 (33)	0.849
Secondary	100 (70)	30 (71)	0.801	75 (56)	50 (54)	0.576
Higher	24 (17)	10 (24)	0.147	6 (5)	5 (5)	0.428
None	0	0	–	10 (7)	7 (8)	0.924
Household head education						
Primary	5 (3)	1 (2)	0.640	16 (12)	12 (13)	0.560
Secondary	74 (52)	16 (38)	**0.035**	50 (37)	30 (33)	0.096
Higher	54 (38)	23 (55)	**0.007**	14 (10)	12 (13)	0.146
Not Applicable	10 (7)	2 (5)	0.500	5 (4)	37 (40)	0.194
None	–	–	–	49 (37)	1 (1)	**0.017**
Occupation						
Unemployed	59 (41)	14 (33)	0.214	90 (67)	60 (65)	0.478
Student	7 (5)	4 (10)	0.098	3 (2)	2 (2)	0.336
Professional	14 (10)	7 (17)	0.074	3 (2)	3 (3)	0.620
Police/Military/Security	5 (4)	3 (7)	0.126	12 (9)	9 (10)	0.137
Trucker/transport business	1 (1)	1 (2)	0.120	1 (1)	2 (2)	–
General worker	6 (4)	2 (5)	0.828	1 (1)	4 (5)	0.318
Self employed	26 (18)	7 (17)	0.762	5 (4)	10 (11)	0.572
Vendor	25 (17)	4 (9)	0.106	16 (12)	2 (2)	0.940
Occupation of household head						
Unemployed	9 (6)	2 (5)	0.627	25 (19)	15 (16)	0.301
Farm worker	1 (1)	0	0.518	2 (1)	2 (2)	0.336
Professional	52 (37)	23 (55)	**0.003**	23 (17)	20 (22)	**0.038**
Police/Military/Security	11 (8)	3 (7)	0.874	5 (4)	5 (5)	0.124
Trucker/transport business	15 (10)	5 (12)	0.722	1 (1)	0	0.137
General worker	5 (4)	1 (2)	0.640	0	0	0.246
Self employed	31 (22)	5 (12)	0.067	30 (22)	18 (20)	0.498
Vendor	7 (5)	0	0.080	1 (1)	1 (1)	0.621
Other	1 (1)	0	0.518	47 (35)	0	–
Not applicable	8 (6)	3 (7)	0.874	0	31 (34)	–
Personal income (US$)						
No income	52 (36)	20 (48)	0.071	77 (57)	52 (57)	0.744
< 200	51 (35)	7 (17)	**0.002**	32 (24)	23 (25)	0.653
200–400	24 (17)	6 (14)	0.606	19 (14)	13 (14)	0.981
430 or more	16 (12)	9 (21)	**0.012**	6 (4)	4 (4)	0.914
Household income (US$)						
No income	52 (36)	21 (50)	**0.029**	71 (53)	50 (55)	0.640
< 600	55 (39)	8 (19)	**0.002**	53 (40)	35 (38)	0.597
600–1000	16 (11)	4 (9)	0.684	6 (4)	4 (4)	0.914
1200 or more	20 (14)	9 (23)	0.098	4 (3)	3 (3)	0.718
Medical insurance/aid						
Yes	50 (35)	25 (60)	**< 0.001**	27 (20)	22 (24)	0.108
No	93 (65)	17 (40)	–	107 (80)	70 (76)	–
Wealth quintiles						
Poorest	50 (35)	28 (67)	**< 0.001**	7 (5)	6 (7)	0.318
Poorer	22 (15)	6 (14)	0.814	32 (24)	23 (25)	0.653
Middle	19 (13)	2 (5)	0.053	36 (27)	23 (25)	0.471
Richer	30 (21)	3 (7)	**0.009**	26 (19)	15 (16)	0.179
Richest	22 (16)	3 (7)	0.078	33 (25)	25 (27)	0.311
Sources of cervical cancer information						
Radio	94 (70)	30 (73)	0.753	31 (25)	23 (27)	0.868
TV	20 (15)	8 (20)	0.747	27 (21)	19 (22)	0.935
Health workers	8 (6)	0	–	57 (45)	36 (42)	0.776
Other	13 (9)	3 (7)	0.911	11 (9)	8 (9)	–
Knowledge that cervical cancer is treatable						
Yes	106 (74)	38 (90)	**0.040**	113 (84)	81 (88)	0.433
No	19 (13)	3 (7)	0.768	7 (5)	2 (2)	0.854
Don’t	18 (13)	1 (3)	0.768	14 (11)	9 (10)	0.934
Number of visits to health facilities or doctors in previous 6 months						
None	46 (32)	4 (10)	0.358	_	_	–
Once	30 (21)	6 (14)	0.695			
Twice	34 (24)	13 (31)	0.624			
Thrice or more	33 (23)	19 (45)	0.099			
Have regular general practitioner (doctor)						
Yes	44 (31)	20 (48)	0.1900.128	_	_	–
No	99 (69)	22 (52)				

Bold shows *p* value < =0.05 indicating statistical significance

There were significant differences in proportions of women who ever screened for cervical cancer and those who never screened (*p* < 0.05) with respect to urban (low and high density) and rural residence, affiliation to protestant, pentecostal and other religions, household heads’ secondary and higher education, household heads’ professional occupation status, income of less than US$200 and US$430 or more, no household income and household income less than US$600, being poorest or richest and knowledge of treatability of cervical cancer. The proportions of cervical cancer patients on treatment and those not treated differed significantly (*p* < 0.05) with respect to high density residence, affiliation to protestant and other religions, household heads with no education, and in professional occupation status.

Table 2 shows the differences in cervical cancer screening and treatment by different socioeconomic groups based on logistic regression model outcomes. Women affiliated to Roman Catholic, Protestant, Pentecostal and Apostolic sect religions were less likely to screen for cervical cancer compared to those in other religions (*p* < 0.05). Women who visited health facilities or consulted doctors once or never six months prior to the survey were less likely to use screening services for cervical cancer (p < 0.05). Age, education, occupation, marital status, medical aid status, income (personal and household), wealth, sources of cervical cancer information, having regular doctor and knowledge of treatability of cervical cancer were not associated with uptake of cervical cancer screening after controlling for household heads’ occupation, education and area of residence. Women with cervical cancer who were affiliated to Protestant churches were 68 times [95% CI: 1.22 to 381] more likely to utilize treatment and care services compared to those in other religions (*p* = 0.040). Province of residence, education, occupation, marital status, income (personal and household), wealth, medical aid status, sources of cervical cancer information and knowledge of treatability of cervical cancer were not associated with utilization of cervical cancer treatment and care after controlling for household heads’ occupation, education and area of residence.

**Table 2:** Multivariate logistic regression analysis showing socio-demographic factors associated with cervical cancer screening of healthy women and treatment of women with cervical cancer

Service type	Screening, *n* = 42			^a^Treatment, *n* = 92		
Socio-demographic variables	OR (adjusted for household head education and occupation)	95% CI	*p*-value	OR (adjusted for household head education, occupation and stage of presentation)	95% CI	*p*-value
Province of residence						
Manicaland	–	–	–	0.57	0.07 to 4.53	0.596
Masvingo	–	–	–	1.86	0.08 to 44.63	0.701
Midlands	–	–	–	0.21	0.01 to 3.97	0.299
Mashonaland Central	–	–	–	0.09	0.00 to 2.35	0.148
Mashonaland East	–	–	–	0.45	0.06 to 3.32	0.439
Mashonaland West	–	–	–	0.22	0.01 to 4.18	0.310
Harare	–	–	–	Ref	–	–
Age (years)						
25–44	0.21	0.02 to 1.90	0.166	0.46	to 13.66	0.653
45 or more	0.29	0.02 to 3.50	0.327	0.95	0.2 to 4.77	0.952
Education						
Primary	0.22	to 895	0.718	0.18	0.00 to 7.54	0.371
Secondary	2.14	0.23 to 19.82	0.500	0.37	to 12.91	0.583
Higher	–	–	–	Ref	–	–
None	Ref	–	–	0.08	0.00 to 4.31	0.212
Occupation						
Unemployed	0.10	0.01 to 1.60	0.103	0.22	0.01 to 5.03	0.347
Professional	0.84	0.05 to 13.11	0.901	0.08	0.00 to 2.19	0.248
Self employed	Ref	–	–	Ref	–	0.681
Other	0.67	0.02 to 22.98	0.826	0.35	0.01 to 21.97	0.507
Marital status						
Married/co-habiting	0.39	to 4.26	0.438	0.14	0.01 to 1.24	0.415
Never married	2.24	0.08 to 63.36	0.637	–	–	–
Widowed	0.09	0.00 to 3.35	0.189	0.46	0.06 to 3.26	0.645
Divorced or separated	Ref	–	–	Ref	–	–
Religion						
Roman Catholic	0.006	0.00 to 0.25	**0.007**	10.60	0.29 to 377	0.195
Protestant	0.01	0.00 to 0.49	**0.020**	68.32	1.22 to 381	**0.040**
Pentecostal	0.003	0.00 to 0.10	**0.002**	16.00	0.46 to 553	0.125
Apostolic sect	0.02	0.00 to 0.93	**0.045**	22.00	0.57 to 846	0.097
Other	Ref	–	–	Ref	–	–
Personal income (US$)						
No income	5.14	0.17 to 151	0.343	0.17	0.00 to 38.26	0.517
< 200	4.47	0.14 to 148	0.401	0.54	0.00 to 80.20	0.808
200–400	1.51	0.07 to 33	0.794	0.57	0.01 to 25.37	0.771
430 or more	Ref	–	–	–	–	–
Household income (US$)						
No income	0.52	0.04 to 6.63	0.618	32.74	to 7017	0.699
< 600	0.13	to 6.23	0.300	5.18	0.06 to 436	0.214
600–1000	0.50	0.02 to 12.41	0.672	0.23	0.01 to 38.23	0.972
1200 or more	Ref	–	–	Ref	–	–
Medical insurance/aid						
Yes	7.24	0.67 to 78.68	0.104	0.84	0.11 to 6.52	0.874
No	Ref			Ref		
Wealth quintiles						
Poor	2.10	0.25 to 17.59	0.492	4.00	0.07 to 227	0.501
Middle	0.04	to 2.05	0.107	0.46	0.07 to 2.95	0.414
Rich	0.23	0.00 to 203	0.799	0.69	0.05 to 9.43	0.781
Sources of cervical cancer information						
Radio	1.18	0.02 to 58.95	0.934	0.16	0.01 to 2.83	0.210
TV	5.48	0.08 to 396	0.436	0.16	0.01 to 3.39	0.236
Health workers	–	–	–	0.26	0.02 to 2.72	0.260
Other	Ref	–	–	Ref	–	–
Knowledge that cervical cancer is treatable						
Yes	4.95	0.21 to 119.27	0.324	1.60	0.21 to 12.10	0.648
No	5.33	to 492.77	0.468	0.02	0.0 to 2.18	0.099
Don’t	Ref	–	–	Ref	–	–
Number of visits to health facilities or doctors in previous 6 months						
None	0.02	0.0 to 0.59	**0.024**	–	–	–
Once	0.08	0.0 to 0.93	**0.044**			
Twice	0.57	0.09 to 3.33	0.537			
Thrice or more	Ref	–	–			
Have regular general practitioner (doctor)						
Yes	0.10	0.01 to 1.82	0.121	–	–	–
No	Ref	–				

Bold shows *p* value < =0.05 indicating statistical significance, ^a^ Treatment included surgery, chemotherapy and radiotherapy as well as palliative chemotherapy or radiation therapy

## Discussion

This study investigated the socio-demographic inequities in uptake of cervical cancer screening and treatment among healthy women and those with cervical cancer aged at least 25 years old. Non-participation in cervical cancer screening was associated with affiliations to Roman Catholic, Protestant, Pentecostal and Apostolic sect religions. Healthy women who never visited health facilities or doctors or only visited once were less likely to screen for cervical cancer. Age, education, occupation, marital status, medical aid status, income (personal and household), wealth, sources of cervical cancer information, having regular doctor and knowledge of treatability of cervical were not associated with uptake of cervical cancer screening services after controlling for confounders. Utilization of treatment for cervical cancer was positively associated with affiliation to Protestant churches. Province of residence, education, occupation, marital status, income (personal and household), wealth, medical aid status, sources of cervical cancer information and knowledge of treatability of cervical cancer were not associated with utilization of cervical cancer treatment and care after controlling for household heads’ occupation, education and area of residence.

The study of socio-demographic inequities of cervical cancer screening and treatment is an important research endeavour in the context of Zimbabwe and other similar contexts. In our study context, inequity is defined as unfair and systematic disparities in the usage of cervical cancer screening and treatment services by groups already disadvantaged with respect to health [34]. We have shown that belonging to major religious affiliations and using health facilities less frequently is associated with low uptake of cervical cancer screening. Over the past few years there have been great strides by the government and its partners to scale-up free screening and treatment of pre-cancerous lesions across the country. A number of screening sites using the VIAC approach have been set up across the country [4, 35]. These facilities are however still centralized at provincial and district levels, although the majority of women are domiciled in rural areas which may be far from service centers [4, 5]. Our work did not find any significant disparities in uptake of cervical cancer screening by urban women compared to their rural counterparts. This finding is not supported by a systematic review conducted in 67 countries where rural residence was a determinant of non-participation in screening for cervical cancer [23]. The National Cervical Cancer Prevention and Control Strategy (2016–2020) was a result of efforts to reduce cervical cancer burden in the country. However, its implementation has remained poor due to limited resources and competing priorities as suggested by recent reports [4, 6]. Despite these efforts, screening uptake has not improved significantly from 2015 when the ZDHS reported 24% [36] while our study found 29% ever screened for cervical cancer in Harare. This suggests low uptake or perhaps underutilization of the cervical cancer screening interventions. While service fees are a major barrier to uptake of health services [7, 11–13], the provision of free screening services was implemented to improve access to all women who may be at risk. Our results suggest that there are potentially additional underlying issues driving low screening utilization.

This research found religious affiliations and usage of health facilities as determinants of participation in cervical cancer screening similar to some earlier work done in Zimbabwe on general health service uptake [11]. In the African context this may suggest the underlying roles of social norms influencing uptake of screening services among healthy women [12, 14, 21, 22, 24]. Some religions, where the majority of women in Zimbabwe are affiliated, influence non-participation in screening services suggesting that belief systems may be important determinants of uptake of preventive interventions [36]. Our findings contrast the results from several other studies done in both developed and developing countries [12–24]. Some Denmark researchers reported basic education, low income and being unmarried as determinants of non-participation in cervical cancer screening services, while our study did not find any associations [15]. Some researchers reported low family disposable income, low education and non-cohabiting influencing non-usage of screening by Swedish women [16] but our work found only religious affiliations and utilization of health facilities as determinants in the context of Harare. A plethora of studies conducted in many countries and at different time points clearly pointed out age, education, income, marital status, employment, family income, ethnicity and wealth as major socio-demographic determinants of inequities to cervical cancer screening [12–24] but our study suggested only religion and usage of health facilities. Findings from another Zimbabwean study [24] were not in agreement with our results suggesting dynamisms in the screening determinants over time and/or other factors influencing cervical cancer screening. However, our work found only two out of the myriad of determinants of screening reported in literature.

While screening for cervical cancer services are fairly increasing in coverage in Zimbabwe, treatment of confirmed cases has remained challenged [4, 6]. We have demonstrated that religion was associated with inequities in the uptake of cervical cancer treatment. Our work found that the majority of socio-demographic factors (age, race, occupation, low socioeconomic class, income, region of residence, health insurance status and wealth) except for religion reported in literature [25–30] were not associated with utilization of cervical cancer treatment in our context. Some studies have reported high costs of treatment for cervical cancer as a major barrier [4, 6, 7], however; we found that income both personal and household as well as wealth were not associated with receipt of treatment. A study in USA found no effect of marital status on receipt of treatment for cervical cancer [29] and this was supported by our work. As opposed to cervical cancer screening which was associated with religious affiliations and usage of health facilities, utilization of treatment for cervical cancer was only associated with one religious affiliation (Protestant) and no other socio-demographic factor reported in literature [25–30]. It is also plausible that inequities in both uptake of screening services and treatment of confirmed cervical cancer in the Zimbabwean context may be potentially entrenched by behavioral, societal and health system factors. These factors may be directly associated with socioeconomic factors. Behavioural factors such as knowledge of cervical cancer, attitudes towards the disease, values concerning health and illness and perceptions of need of routine screening and treatment may be important to meditate on. Societal factors may include social norms about routine screening and treatment, technologies, social networks/support for screening and treatment uptake, beliefs about cervical cancer, myths/misconceptions/taboos about the disease and its treatment. There are a plethora of potential health system determinants and these may include: number and distribution of screening and treating centres, health worker knowledge and skills, organization of health service delivery, physical infrastructure, policies and supportive laws [37, 38]. Further research work involving mixed method designs is imperative to fully understand the major drivers of inequities in low-income settings.

The strengths of our study lie in several aspects, mainly that a robust cross sectional design was conducted. The design involved stratification of known confounding variables i.e. area of residence and socioeconomic classification in the surveys. The patient survey was based in tertiary health facilities providing treatment and care for cervical cancer in Zimbabwe. The use of multivariate analyses allowed for meaningful associations to be identified given that confounding variables were adjusted for. More importantly, this study used a structured questionnaire that we designed and validated for use in developing country context [33]. In addition this study improved the methods of other studies cited in that it had two groups of participants, healthy women and those with cervical cancer in different study settings and comparisons of the factors associated with screening and treatment at the same time point gave a better picture of the status of wider cervical cancer interventions in the country. This makes the findings of this research a good starting point in improving policy and programmes. However, this research being a cross sectional design prevented causal inferences to be established. Furthermore, cervical cancer screening status was self-reported from healthy women who had been selected randomly in the communities. Self-reported data may be biased as there is no means of verification. However, our validated tool [33] had follow-up questions on “*Last date of screening*” and “*Name of health facility where last screening was conducted*” in order to validate the screening status. For the patient survey, only participants from public health facilities and an NGO facility were recruited as two private health facilities providing cancer treatment declined to approve this study. However; the majority of cervical cancer patients in Zimbabwe are treated in public health facilities and the inherent bias of conducting this study in these facilities was minimal. Small sample sizes can also compromise statistical precision.

## Conclusion

Our study revealed few variations in the participation of women in cervical cancer screening explained by religious affiliations and usage of health facilities. While our results suggested that receipt of cervical cancer treatment was only associated with protestant religious affiliation there may be indirect relationship through disease stage at presentation which in turn influences treatment modalities. There is need to strengthen health education in communities including churches to improve understanding of cervical cancer in an endeavour to increase uptake of screening and treatment services. Strengthening of universal coverage of healthcare services in our low-income context is also recommended. The design of targeted interventions to improve participation in preventive measures and usage of treatment services for cervical cancer is implied.

## Abbreviations

AIDS: Acquired immunodeficiency syndrome
HIV: Human immunodeficiency virus
HPV: Human papilloma virus
NGO: Non-governmental organization
VIAC: Visual inspection with acetic acid cervicography
ZDHS: Zimbabwe demographic and health survey
